# Common Effects of Amnestic Mild Cognitive Impairment on Resting-State Connectivity Across Four Independent Studies

**DOI:** 10.3389/fnagi.2015.00242

**Published:** 2015-12-24

**Authors:** Angela Tam, Christian Dansereau, AmanPreet Badhwar, Pierre Orban, Sylvie Belleville, Howard Chertkow, Alain Dagher, Alexandru Hanganu, Oury Monchi, Pedro Rosa-Neto, Amir Shmuel, Seqian Wang, John Breitner, Pierre Bellec, for the Alzheimer's Disease Neuroimaging Initiative

**Affiliations:** ^1^McGill UniversityMontreal, QC, Canada; ^2^Douglas Mental Health University Institute, Research CentreMontreal, QC, Canada; ^3^Centre de Recherche de L'institut Universitaire de Gériatrie de MontréalMontreal, QC, Canada; ^4^Université de MontréalMontreal, QC, Canada; ^5^University of CalgaryCalgary, AB, Canada; ^6^Hotchkiss Brain InstituteCalgary, AB, Canada

**Keywords:** fMRI, mild cognitive impairment, connectome, resting-state, default mode network, meta-analysis

## Abstract

Resting-state functional connectivity is a promising biomarker for Alzheimer's disease. However, previous resting-state functional magnetic resonance imaging studies in Alzheimer's disease and amnestic mild cognitive impairment (aMCI) have shown limited reproducibility as they have had small sample sizes and substantial variation in study protocol. We sought to identify functional brain networks and connections that could consistently discriminate normal aging from aMCI despite variations in scanner manufacturer, imaging protocol, and diagnostic procedure. We therefore combined four datasets collected independently, including 112 healthy controls and 143 patients with aMCI. We systematically tested multiple brain connections for associations with aMCI using a weighted average routinely used in meta-analyses. The largest effects involved the superior medial frontal cortex (including the anterior cingulate), dorsomedial prefrontal cortex, striatum, and middle temporal lobe. Compared with controls, patients with aMCI exhibited significantly decreased connectivity between default mode network nodes and between regions of the cortico-striatal-thalamic loop. Despite the heterogeneity of methods among the four datasets, we identified common aMCI-related connectivity changes with small to medium effect sizes and sample size estimates recommending a minimum of 140 to upwards of 600 total subjects to achieve adequate statistical power in the context of a multisite study with 5–10 scanning sites and about 10 subjects per group and per site. If our findings can be replicated and associated with other established biomarkers of Alzheimer's disease (e.g., amyloid and tau quantification), then these functional connections may be promising candidate biomarkers for Alzheimer's disease.

## Introduction

Resting-state connectivity in functional magnetic resonance imaging (fMRI) captures the spatial coherence of spontaneous fluctuations in blood oxygenation. Resting-state fMRI is a promising technique that may be useful as an early biomarker for Alzheimer's disease (AD), a neurodegenerative process that develops over decades before patients suffer from dementia. The possibility that disturbed resting-state connectivity may be an early marker for AD is supported by studies of mild cognitive impairment (MCI), a disorder characterized by objective cognitive deficits without dementia, i.e., without impairment in activities of daily living, and more specifically by studies of amnestic MCI (aMCI), the most common subtype of MCI characterized by memory deficits (Petersen et al., 2001). These studies showed altered functional connectivity in MCI compared with cognitively normal elderly (CN; Sorg et al., 2007; Bai et al., 2009; Liang et al., 2012; Wu et al., 2014), but they relied on small sample sizes (*n* = ~40) and differed in many aspects of their protocols, e.g., recruitment and image acquisition procedures. If resting-state fMRI is to serve as a useful biomarker of AD, or any pathology, for clinical practice or research, we must determine if changes in functional connectivity differences between groups of subjects are robust to such variation in study protocols. Therefore, we sought to identify brain connections that showed consistent MCI-related changes across multiple independent studies. If such connections exist, they may be used as targets to be examined alongside other established AD biomarkers (e.g., amyloid and tau measures) in order to validate resting-state fMRI's potential as a biomarker for AD.

Resting-state connectivity studies have consistently found decreased connectivity between nodes within the default mode network (DMN) in patients with AD or MCI compared with CN (Sorg et al., 2007; Bai et al., 2009; Zhang et al., 2010; Koch et al., 2012; Liang et al., 2012). Less consistent are reports of alterations in the executive attentional, frontoparietal, and anterior temporal networks (Sorg et al., 2007; Zhang et al., 2010; Gour et al., 2011; Agosta et al., 2012; Liang et al., 2012; Wu et al., 2014) due to the literature's bias toward investigating the DMN. Further inconsistencies can be found in some studies that have reported increased connectivity between the middle temporal lobe and other DMN areas in MCI (Qi et al., 2010), while others have reported decreased connectivity between these same regions (Bai et al., 2009) and others have reported no significant differences between MCI and CN (Koch et al., 2012).

One obvious explanation for such inconsistency may be these studies' small sample sizes resulting in low statistical power (Kelly et al., 2012). Beyond this, however, there are other methodological differences that may compromise the comparison of results across independent studies. For example, the criteria for recruiting subjects with MCI, e.g., Petersen (2004) vs. NIA-AA recommendations (Albert et al., 2011) may differ among studies. Different study samples may also reflect different socio-cultural characteristics of recruiting sites, e.g., ethnicity, language, diet, socioeconomic status. The fMRI measurements themselves can also be affected by differences in details of the image acquisition such as scanner make and model (Friedman et al., 2006), sequence parameters such as repetition time, flip angle, or acquisition volume (Friedman and Glover, 2006), experimental design such as eyes-open/eyes-closed (Yan et al., 2009) or experiment duration (van Dijk et al., 2010), and scanning environment such as sound attenuation measures (Elliott et al., 1999), room temperature (Vanhoutte et al., 2006), or head-motion restraint techniques (Edward et al., 2000).

To identify robust changes in resting-state connectivity between aMCI and CN, we implemented a meta-analysis of four independent resting-state fMRI datasets (ADNI2 and three small single-site studies) using a weighted average implemented by Willer et al. (2010). Rather than relying on a priori target regions or connections, we leveraged the large sample size to perform a systematic search of brain connections affected by aMCI, an approach termed a “connectome-wide association study” (Shehzad et al., 2014). In addition, we relied on functionally-defined brain parcellations using an automated clustering procedure and we explored the impact of the number of brain clusters (called resolution) on observed differences (Bellec et al., 2015).

## Methods

### Participants

We combined data from four independent studies: the Alzheimer's Disease Neuroimaging Initiative 2 (ADNI2) sample, two samples from the Centre de recherche de l'institut universitaire de gériatrie de Montréal (CRIUGMa and CRIUGMb), and a sample from the Montreal Neurological Institute (MNI; Wu et al., 2014). All participants gave their written informed consent to engage in these studies, which were approved by the research ethics board of the respective institutions, and included consent for data sharing with collaborators as well as secondary analysis. Ethical approval was also obtained at the site of secondary analysis (CRIUGM).

The ADNI2 data used in the preparation of this article were obtained from the Alzheimer's Disease Neuroimaging Initiative (ADNI) database (adni.loni.usc.edu). ADNI was launched in 2003 by the National Institute on Aging, the National Institute of Biomedical Imaging and Bioengineering, the Food and Drug Administration, private pharmaceutical companies and non-profit organizations, as a $60 million, 5-year public-private partnership representing efforts of co-investigators from numerous academic institutions and private corporations. ADNI was followed by ADNI-GO and ADNI-2 that included newer techniques. Subjects included in this study were recruited by ADNI-2 from all 13 sites that acquired resting-state fMRI on Philips scanners across North America. For up-to-date information, see www.adni-info.org.

The combined sample included 112 CN and 143 aMCI prior to quality control. After quality control, 99 CN and 129 aMCI remained. In the CN group, the mean age was 72.0 (*s.d*. = 7.0) years, and 37% were men. Mean age of the aMCI subjects was 72.3 (*s.d*. = 7.6) years, and 50% were men. An independent samples *t*-test did not reveal any significant difference in age between the groups (*t* = 0.759, *p* = 0.448). A chi-squared test revealed a trend toward a significant difference in gender distribution between the groups (χ^2^ = 3.627, *p* = 0.057). Note that both age and gender were entered as confounding variables in the statistical analysis below. See Table 1 for sample size and demographic information from the individual studies after passing quality control (for information about the original cohorts before quality control, see Supplementary Table 1).

**Table 1:** **Demographic information in all studies after quality control**.

		**ADNI2**	**CRIUGMa**	**CRIUGMb**	**MNI**	**Combined sample**
**CN**	**N**	**49**	**18**	**17**	**15**	**99**
	Mean age (*s.d*.)	74.4 (6.8)	71.2 (8.0)	70.4 (4.6)	67.0 (5.7)	72.0 (7.0)
	Number male (%)	21 (43%)	7 (39%)	2 (12%)	7 (47%)	37 (37%)
	Mean years of education (*s.d*.)^a^	16.9 (2.2)	14.9 (2.3)	15.1 (2.8)	15.0 (3.1)	16.0 (2.6)
	MMSE mean (range)	28.7 (25–30)	28.8 (27–30)	n/a	29.0 (27–30)	n/a
	MoCA mean (range)	n/a	27.8 (22–30)	28.4 (26–30)	n/a	n/a
**aMCI**	**N**	**82**	**8**	**21**	**18**	**129**
	Mean age (*s.d*.)	71.2 (7.3)	79.9 (6.1)	74.8 (7.0)	71.2 (8.1)	72.3 (7.6)
	Number male (%)	43 (52%)	3 (38%)	12 (57%)	7 (39%)	65 (50%)
	Mean years of education (*s.d*.)^a^	16.2 (2.6)	13.7 (3.8)	14.8 (4.2)	13.1 (3.1)	15.5 (3.2)
	MMSE mean (range)	28.1 (24–30)^*^	26.1 (22–29)^*^	n/a	26.1 (22–30)^*^	n/a
	MoCA mean (range)	n/a	23.3 (20–29)^*^	24.6 (16–29)^*^	n/a	n/a

*MMSE, Mini-mental state examination; MoCA, Montreal Cognitive Assessment*.

* *Significant difference between aMCI and CN (within study) for independent samples t-test at p ≤ 0.05*.

a *Missing values for education for subjects in ADNI2 (1 CN, 1 aMCI), CRIUGMb (2 aMCI), and MNI (3 CN, 6 aMCI)*.

All subjects underwent cognitive testing (e.g., memory, language, and executive function; see Table 2 for a list of specific tests used in each study). Exclusion criteria common to all studies included: Contraindications to MRI, presence or history of axis I psychiatric disorders (e.g., depression, bipolar disorder, schizophrenia), presence or history of neurologic disease with potential impact on cognition (e.g., Parkinson's disease), and presence or history of substance abuse. CN subjects could not meet criteria for MCI or dementia. Those with aMCI had memory complaints, objective cognitive loss (based on neuropsychological testing), but had intact functional abilities and did not meet criteria for dementia. In ADNI2, the diagnosis of aMCI was made based on an education adjusted abnormal score on the Logical Memory II subscale (Delayed Paragraph Recall, Paragraph A only) from the Wechsler Memory Scale and a Clinical Dementia Rating (CDR) of 0.5. In both CRIUGMa and CRIUGMb, the diagnosis of aMCI was made based on scores equal to or >1.5 standard deviations below the mean adjusted for age and education on memory tests. At the MNI, the diagnosis of aMCI relied on the Petersen criteria (2004). At both CRIUGMb and MNI, aMCI diagnoses were made with input from a neurologist. See the Supplementary Methods (Datasheet 1 in Supplementary Material) for greater details for each study.

**Table 2:** **Neuropsychological tests that were used in each study**.

**Test**	**ADNI2**	**CRIUGMa**	**CRIUGMb**	**MNI**
Mini-mental state examination (MMSE)	x	x		x
Montreal Cognitive Assessment (MoCA)	x	x	x	
Clinical Dementia Rating (CDR)	x		x	
ADAS-Cog	x			
Everyday Cognition (ECog)	x			
Trail making	x	x	x	x
	(Trails A and B)	(Trails A and B)	(Trails A and B)	(DKEFS)
Boston naming test	x	x	x	x
Digit span		x	x	x
Color-word interference (DKEFS)		x	x	x
Rey auditory verbal learning test	x	x		x
Verbal fluency	x	x	x (MEC)	x (DKEFS)
Clock drawing	x	x		
Visual object and space perception battery		x		
Brixton spatial anticipation test			x	
Hooper visual organization test			x	
Rey complex figure		x	x	x
Aggie figures learning test				x
16-Item free and cued recall (RL/RI-16)			x	
Pyramid and palm trees test		x		
Weschler memory scale—logical memory subtest	x	x	x	

*MEC, Montréal évaluation de la communication; DKEFS, Delis–Kaplan Executive Function System*.

### Imaging data acquisition

All resting-state fMRI and structural scans were acquired on 3T scanners. We performed analyses on the first usable scan (typically the baseline scan) from ADNI2 and applied clinical diagnoses from the same study time point as the first usable scan for each participant in that dataset. See Table 3 for acquisition parameters for each sample.

**Table 3:** **Structural and functional scan acquisition parameters**.

	**ADNI2^a^**	**CRIUGMa**	**CRIUGMb**	**MNI**
**Scanner manufacturer**	**Philips**	**Siemens**	**Siemens**	**Siemens**
**STRUCTURAL**				
No. channels	8	32	32	32
No. slices	170	176	176	176
Slice thickness (mm)	1.2	1	1	1
In-plane resolution (mm × mm)	1 × 1	1 × 1	1 × 1	1 × 1
Matrix size	256 × 256	240 × 256	256 × 256	256 × 256
FOV (mm^2^)	256	240/256	256	256
TR (s)	6.8	2.3	2.53	2.3
TE (ms)	3.09	2.91	1.64	2.98
TI (s)	n/a	0.9	1.2	0.9
FA (°)	9	9	7	9
Slice gap	0	0	0	0
Imaging plane	Sagittal	Sagittal	Sagittal	Sagittal
NEX	1	1	1	1
**FUNCTIONAL**				
No. runs	1	1	3	3
No. channels	8	32	32	32
No. volumes	140	240	150	160
No. slices	48	33	42	38
Slice thickness (mm)	3.3	4	3.4	3.6
In-plane resolution (mm × mm)	3.3 × 3.3	3 × 3	3.4 × 3.4	3.6 × 3.6
Matrix size	64 × 64	64 × 64	64 × 64	64 × 64
FOV (mm^2^)	212	192	218	230
TR (s)	3	2	2.6	2
TE (ms)	30	30	30	30
FA (°)	80	90	90	90
Slice gap	0	0	0	0
Imaging plane	Axial	Axial	Axial	Axial
NEX	1	1	1	1
Total scan time (min:s)	7:00	8:00	19:30	16:00

a *http://adni.loni.usc.edu/wp-content/uploads/2011/04/ADNI_3T_Philips_2.6.pdf*.

### Computational environment

All experiments were performed using the NeuroImaging Analysis Kit (NIAK^1^; Bellec et al., 2011) version 0.12.18, under CentOS version 6.3 with Octave^2^ version 3.8.1 and the Minc toolkit^3^ version 0.3.18. Analyses were executed in parallel on the “Guillimin” supercomputer^4^, using the pipeline system for Octave and Matlab (Bellec et al., 2012), version 1.0.2. The scripts used for processing can be found on Github^5^.

### Pre-processing

Each fMRI dataset was corrected for slice timing; a rigid-body motion was then estimated for each time frame, both within and between runs, as well as between one fMRI run and the T1 scan for each subject (Collins and Evans, 1997). The T1 scan was itself non-linearly co-registered to the Montreal Neurological Institute (MNI) ICBM152 stereotaxic symmetric template (Fonov et al., 2011), using the CIVET pipeline (Ad-Dab'bagh et al., 2006). The rigid-body, fMRI-to-T1 and T1-to-stereotaxic transformations were all combined to resample the fMRI in MNI space at a 3 mm isotropic resolution. To minimize artifacts due to excessive motion, all time frames showing a displacement >0.5 mm were removed (Power et al., 2012). A minimum of 50 unscrubbed volumes per run was required for further analysis (13 CN and 14 aMCI were rejected from the original cohort of 112 CN and 143 aMCI). Neither the rate of rejection nor the frame displacement values (before and after scrubbing) varied significantly among the four samples or between CN and aMCI. The following nuisance covariates were regressed out from fMRI time series: slow time drifts (basis of discrete cosines with a 0.01 Hz high-pass cut-off), average signals in conservative masks of the white matter and the lateral ventricles as well as the first 3–10 principal components (median numbers for ADNI2, CRIUGMa, CRIUGMb, and MNI were 9, 6, 7, and 7, respectively, and accounting for 95% variance) of the six rigid-body motion parameters and their squares (Lund et al., 2006; Giove et al., 2009). The fMRI volumes were finally spatially smoothed with a 6 mm isotropic Gaussian blurring kernel. A more detailed description of the pipeline can be found on the NIAK website^6^ and Github^7^.

### Bootstrap analysis of stable clusters (BASC)

We applied a BASC to identify clusters that consistently exhibited similar spontaneous BOLD fluctuations in individual subjects, and were spatially stable across subjects. We first applied a region-growing algorithm to reduce each fMRI dataset into a time × space array, with 957 regions (Bellec et al., 2006). BASC replicates a hierarchical Ward clustering 1000 times and computes the probability that a pair of regions fall in the same cluster, a measure called stability. The region × region stability matrix is fed into a clustering procedure to derive consensus clusters, which are composed of regions with a high average probability of being assigned to the same cluster across all replications. At the individual level, the clustering was applied to the similarity of regional time series, which was replicated using a circular block bootstrap. Consensus clustering was applied to the average individual stability matrix to identify group clusters. The group clustering was replicated via bootstrapping of subjects in the group. A consensus clustering was finally applied on the group stability matrix to generate group consensus clusters.

The cluster procedure was carried out at a specific number of clusters (called resolution). Using a “multiscale stepwise selection” (MSTEPS) method (Bellec, 2013), we determined a subset of resolutions that provided an accurate summary of the group stability matrices generated over a fine grid of resolutions: 4, 6, 12, 22, 33, 65, 111, and 208.

### Derivation of functional connectomes

**Figure 1:**
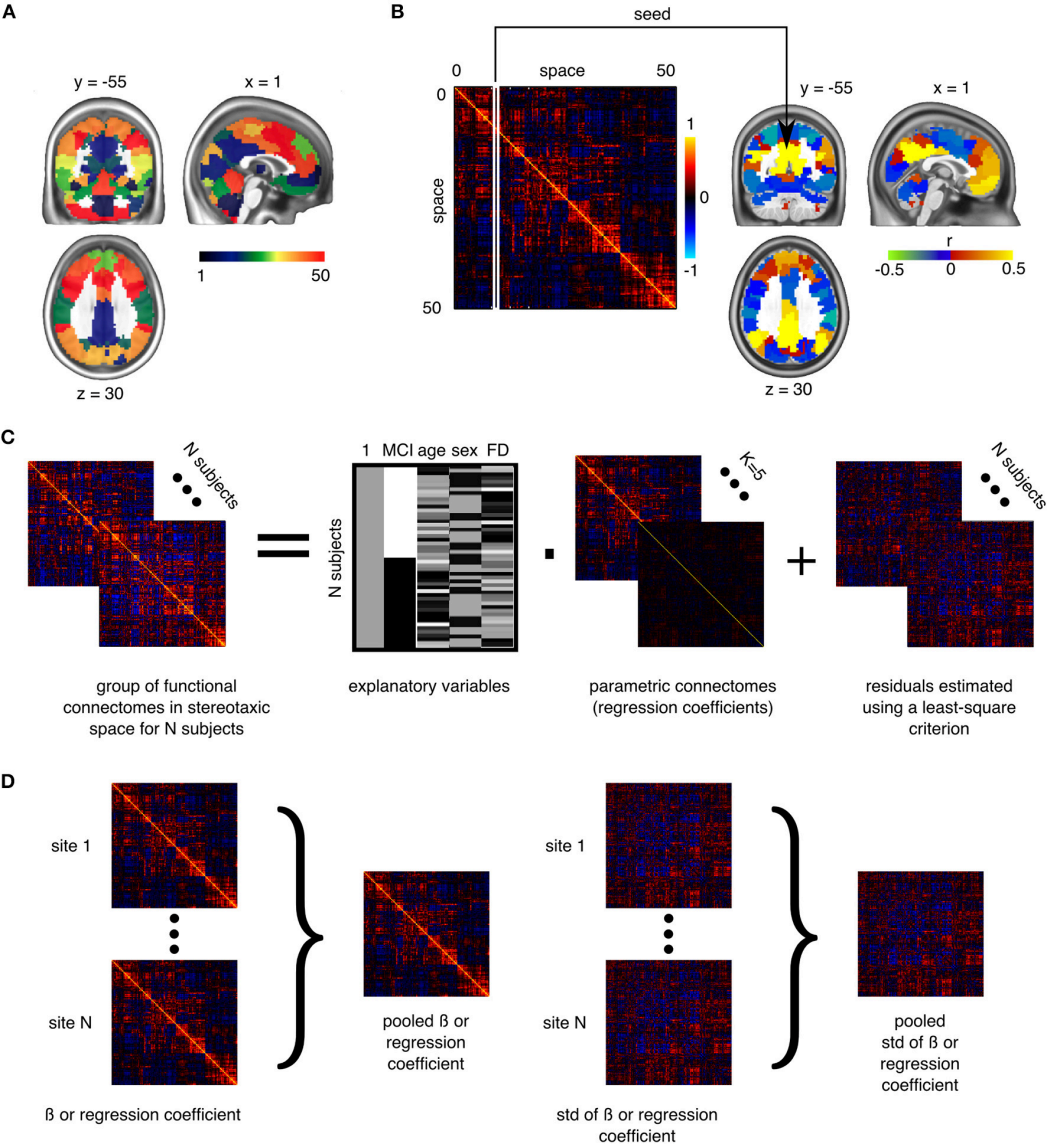
**Application of general linear models to connectomes**. **(A)** The brain is functionally parcellated into *K* (e.g., 50) clusters generated through a clustering algorithm. **(B)** The connectome is a *K* × *K* matrix measuring functional connectivity between and within clusters. **(C)** A general linear model is used to test the association between phenotypes and connectomes, independently at each connection, at the group level. **(D)** In a multisite situation, independent site-specific effects are estimated and then pooled through weighted averaging (Willer et al., 2010).

For each resolution *K*, and each pair of distinct clusters, the between-clusters connectivity was measured by the Fisher transform of the Pearson's correlation between the average time series of the clusters. The within-cluster connectivity was the Fisher transform of the average correlation between time series inside the cluster. An individual connectome was thus a *K* × *K* matrix. See Figures 1A,B for an illustration of a parcellation and associated connectome.

### Statistical testing

To test for differences between aMCI and CN at a given resolution, we used a general linear model (GLM) for each connection between two clusters. The GLM included an intercept, the age and sex of participants, and the average frame displacement of the runs involved in the analysis. The contrast of interest (aMCI–CN) was represented by a dummy covariate coding the difference in average connectivity between the two groups. All covariates except the intercept were corrected to a zero mean (Figure 1C). The GLM was estimated independently for each scanning protocol. In addition to distinguishing between CRIUGMa, CRIUGMb, MNI, and ADNI2, ADNI2 was subdivided into five sub-studies based on the use of different Philips scanner models (i.e., Achieva, Gemini, Ingenia, Ingenuity, and Intera). We dropped all subjects scanned with Ingenuity (2 CN, 1 aMCI) due to the elimination of all aMCI subjects within that site by the scrubbing procedure and its small sample size. We therefore estimated seven independent GLMs for each protocol (ADNI2-Achieva, ADNI2-Gemini, ADNI2-Ingenia, ADNI2-Intera, CRIUGMa, CRIUGMb, MNI). The estimated effects were combined across all protocols through inverse variance based weighted averaging (Willer et al., 2010; Figure 1D).

Resolutions containing fewer than 50 clusters have been suggested to have higher sensitivity based on prior independent work (Bellec et al., 2015). The GLM was first applied at an a priori resolution of *K* = 33, which was the lowest number of clusters for which the DMN could be clearly decomposed into subnetworks (Supplementary Figure 1, visit Figshare for 3D volumes of brain parcellations^8^ and see Supplementary Table 2 for a list of the 33 clusters and their numerical IDs). The false-discovery rate (FDR) across connections was controlled at *q*^*FDR*^ ≤ 0.1 (Benjamini and Hochberg, 1995). In addition to the analysis at resolution 33, we assessed the impact of that parameter by replicating the GLM analysis at the seven resolutions selected by MSTEPS (Supplementary Figure 2). We implemented an omnibus test (family-wise error rate α ≤ 0.05) to assess the overall presence of significant differences between groups, pooling FDR results across all resolutions (Bellec et al., 2015). If the omnibus test across resolutions was not significant, then no test would be deemed significant. Since this omnibus test was significant, we used the FDR threshold of *q* ≤ 0.1 to explore single resolutions.

## Results

### Functional connectivity differences between aMCI and CN

**Figure 2:**
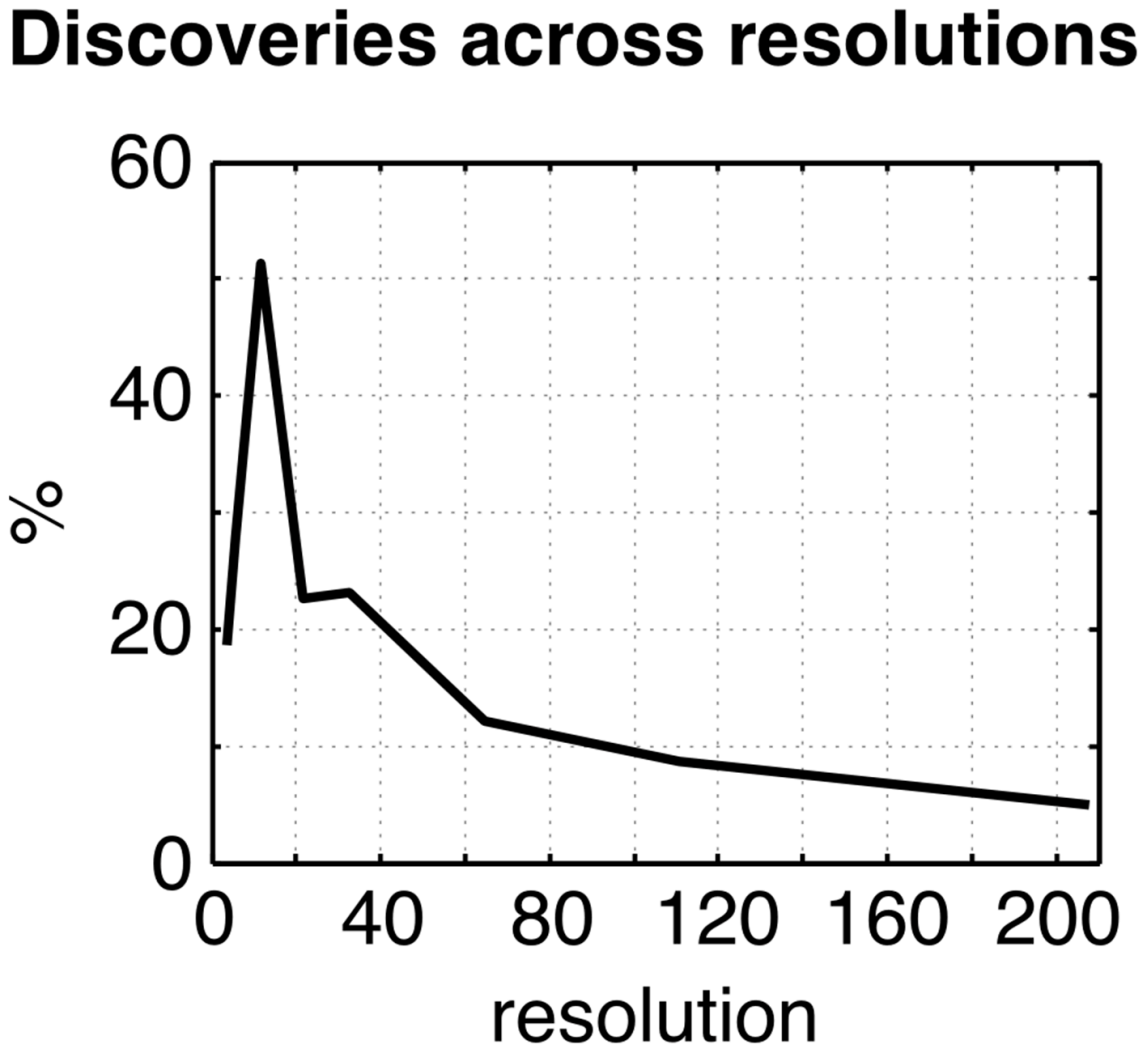
**Plot of the percentage of connections identified as significant by the statistical comparison between aMCI and CN across the connectome (***q***^***FDR***^ ≤ 0.1), as a function of the resolutions selected by MSTEPS**.

**Figure 3:**
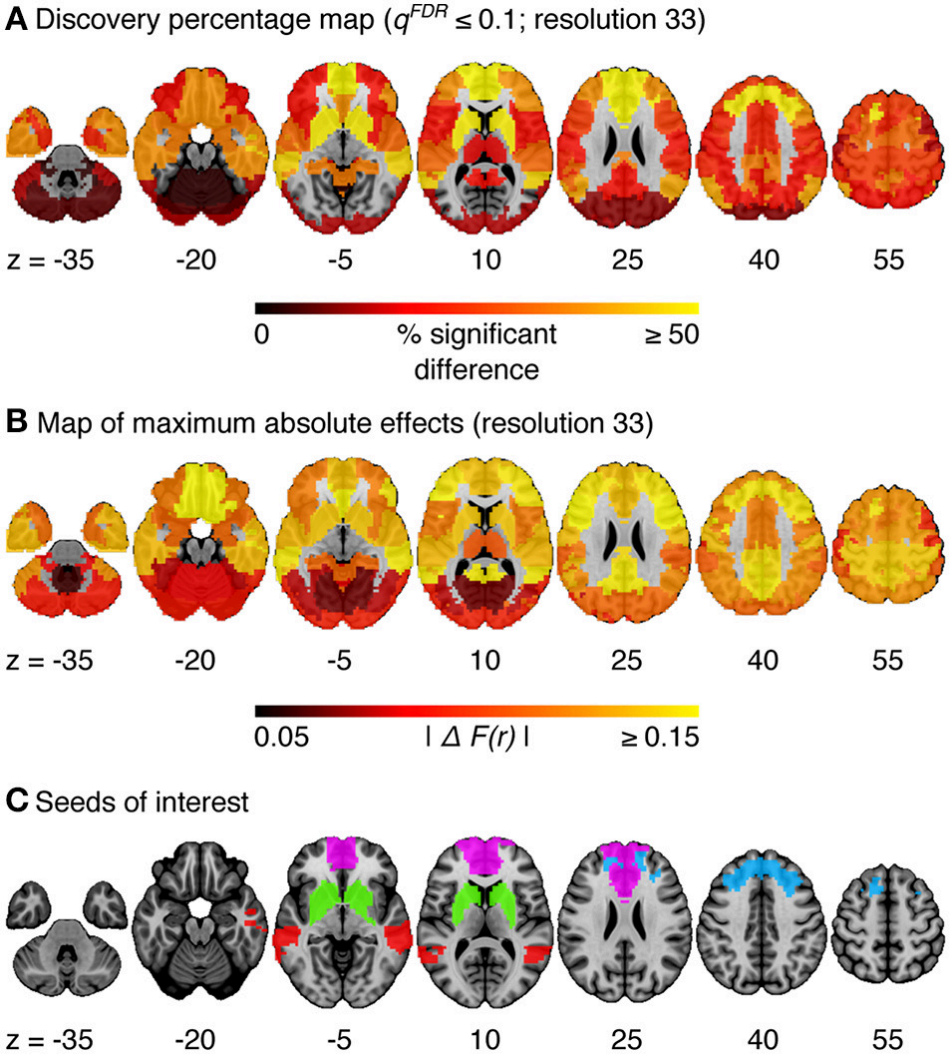
**(A)** Map of the percentage of connections associated with a given cluster and identified as significant by the statistical comparison between aMCI and CN, at a resolution of 33 clusters (*q*^*FDR*^ ≤ 0.1). **(B)** Maximum absolute difference in average connectivity between aMCI and CN, across all connections associated with a cluster, at resolution 33. ΔF(r) signifies the difference in Fisher-transformed correlation values between the groups. **(C)** Four clusters of interest (superior medial frontal cortex, dorsomedial prefrontal cortex, striatum, middle temporal lobe) were selected out of 33 for further characterization.

**Figure 4:**
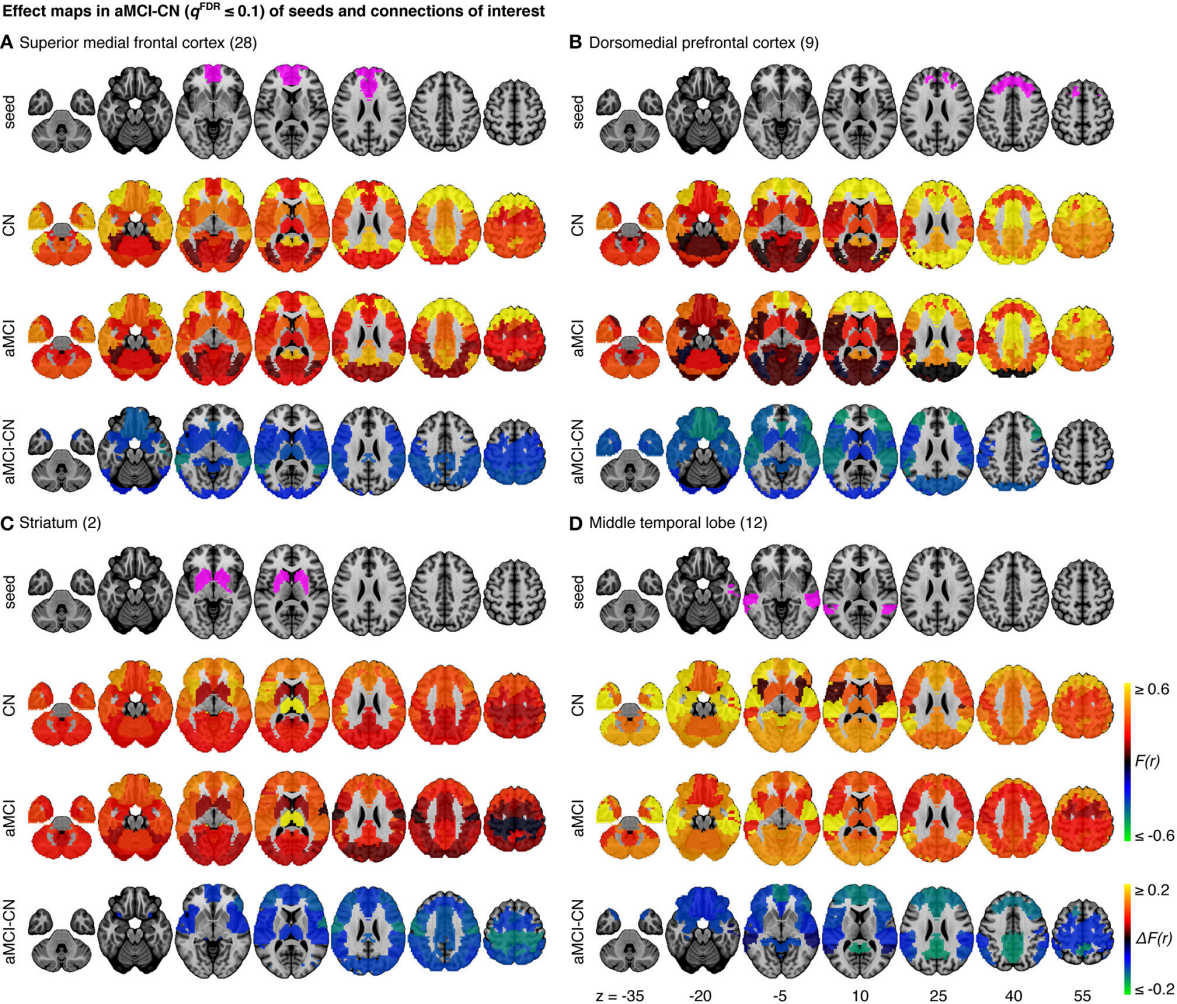
**Effect maps for a selection of four seeds that show effects related to aMCI at resolution 33**. Effect maps reveal the spatial distribution of the changes in functional connectivity for **(A)** the superior medial frontal cortex, **(B)** the dorsomedial prefrontal cortex, **(C)** striatum, and **(D)** the middle temporal lobe. All connections shown in the maps of difference in average connectivity between aMCI and CN are significant at *q*^*FDR*^ ≤ 0.1. For each panel, the top line maps the spatial location of the seed region in magenta, the second and third lines show the connectivity (Fisher-transformed correlation values, *F(r)*) between the designated seed region and the rest of the brain in CN and aMCI, respectively, and the fourth line shows a difference map between aMCI and CN [difference in Fisher-transformed correlation values, Δ*F(r)*]. The numbers in parentheses refer to the numerical IDs of the clusters in the 3D parcellation volume, as listed in Supplementary Table 2.

The omnibus test pooling significant differences in connectivity between aMCI and CN across all resolutions was significant at α ≤ 0.05 (*p* ≤ 0.0056). In line with prior observations on independent datasets (Bellec et al., 2015), resolutions containing fewer than 50 clusters were associated with a higher rate of discovery (Figure 2). At resolution 33, significant group differences between aMCI and CN were seen across the whole brain (Figure 3A). Four brain clusters were associated with 47% of all significant changes found across the connectome: the superior medial frontal cortex (including anterior cingulate), dorsomedial prefrontal cortex, striatum, and middle temporal lobe (Figures 3B,C, Supplementary Table 3). Supplementary Table 3 contains a list of parcels that account for all non-redundant significant connectivity differences between aMCI and CN. For example, the first-ranked seed (superior medial frontal cortex) was associated with 13.4% of connections that differ between the groups. The second-ranked seed (dorsomedial prefrontal cortex) was associated with an additional 12.7% of connectivity differences that did not overlap with or were not previously accounted for by the first seed. Note that if a given parcel was associated with a significant effect with another region that ranked in the table, then that parcel may not be listed in the table (i.e., this table is not a comprehensive list of parcels that show significant effects, as a given parcel may involve a region in the table at a higher rank which already accounted for its effects). Given that the top four clusters explained nearly half of the findings, they were further characterized in seed-based connectivity analyses, which revealed that aMCI showed decreased connectivity between DMN nodes and between areas of the cortico-striatal-thalamic loop (Figure 4). More specifically, in aMCI compared to CN, the superior medial frontal cortex displayed significantly reduced connectivity with the ventromedial prefrontal cortex, striatum, thalamus, temporal lobes, hippocampus, inferior parietal lobes, and precuneus (Figure 4A). aMCI showed reduced connectivity between the dorsomedial prefrontal cortex with temporal lobe regions, ventral frontal areas, thalamus, striatum, and the cuneus (Figure 4B). The striatum in aMCI also exhibited decreased connectivity with the sensorimotor cortex, thalamus, and frontal and parietal regions (Figure 4C). Lastly, in aMCI, the middle temporal lobe displayed significantly decreased connectivity with the posterior cingulate, precuneus, inferior parietal lobes, hippocampus, and frontal areas (Figure 4D).

### Sample-specific effects

**Figure 5:**
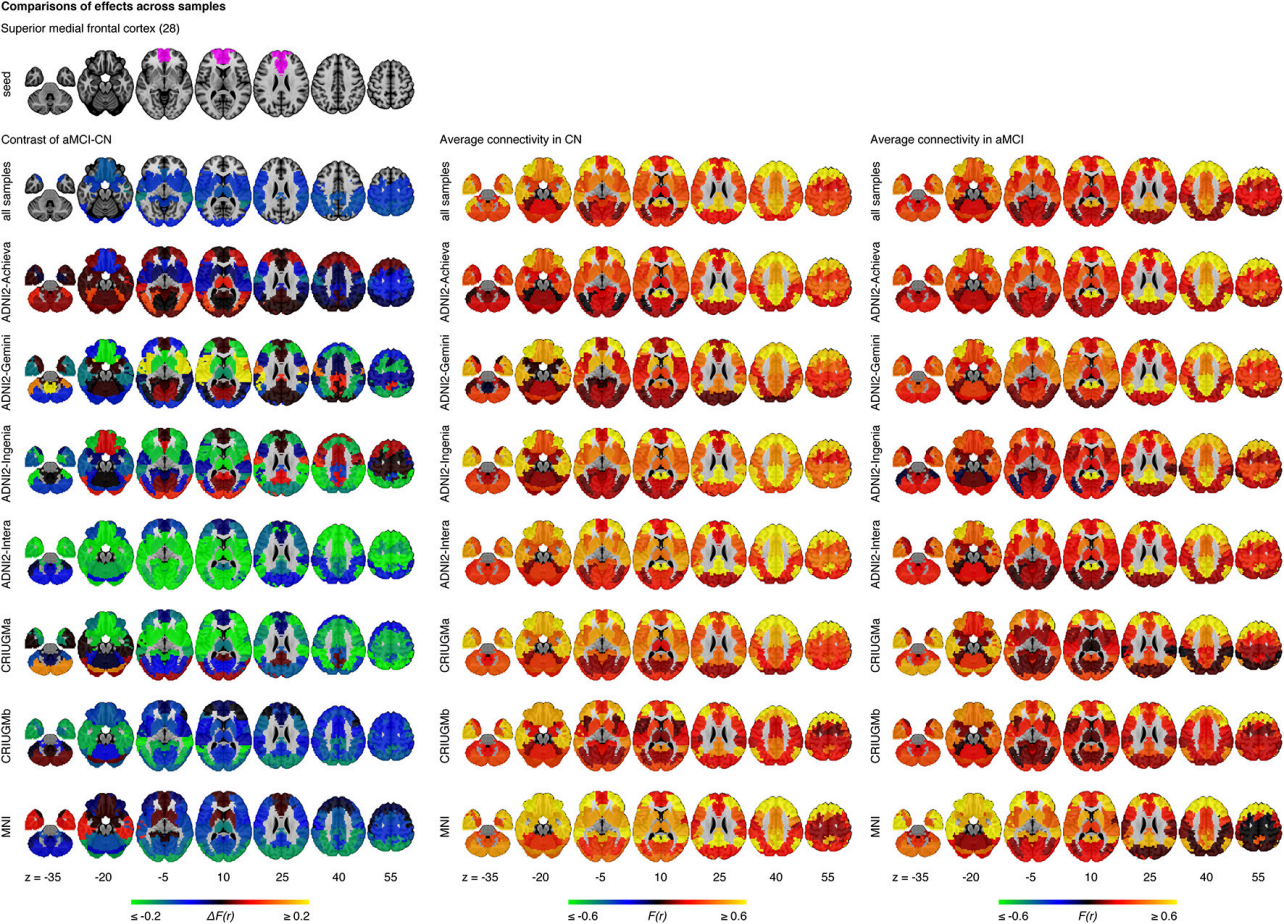
**Comparisons of effects in the superior medial frontal cortex across samples**. This figure illustrates functional connectivity changes between aMCI and CN, average connectivity in CN, and average connectivity in aMCI in each site (ADNI2-Achieva, ADNI2-Gemini, ADNI2-Ingenia, ADNI2-Intera, CRIUGMa, CRIUGMb, MNI) independently of other sites and when samples are pooled together (all samples). The number in parentheses refers to the numerical ID of the seed in the 3D parcellation volume, as listed in Supplementary Table 2.

The statistical model we used to combine GLM analyses across sites was based on a weighted average. The possibility thus existed that an effect would be significant in the pooled analysis because it was driven by a very strong effect in a single sample, instead of being consistent across all samples. When we examined effects in each sample independently, we detected no findings or very few significant findings. We then explored the whole brain connectivity of the top four seed regions (superior medial frontal cortex, dorsomedial prefrontal cortex, striatum, and middle temporal lobe) within each sample. The majority of effects found at each sample did not appear to be consistent or reproducible across studies as the comparison between aMCI and CN varied substantially among the seven samples (Figure 5, Supplementary Figures 3–5). We assessed the extent at which findings among the seven samples were similar by calculating correlation coefficients across the spatial maps for the average connectivity values in CN, the average connectivity values in aMCI, and differences in connectivity values between aMCI and CN among the samples. We found that the difference maps, contrasting aMCI and CN, were weakly correlated on average across studies and protocols (mean *r* = 0.06, min *r* = −0.64, max *r* = 0.69). The average connectivity maps among studies in both CN and aMCI were generally highly correlated with each other (for CN, mean *r* = 0.68, min *r* = −0.16, max *r* = 0.95; for aMCI, mean *r* = 0.67, min *r* = −0.10, max *r* = 0.97). These results were expected given the small sample sizes of most independent samples (Kelly et al., 2012), but still sobering as the majority of the literature on aMCI and fMRI has used small sample sizes.

**Figure 6:**
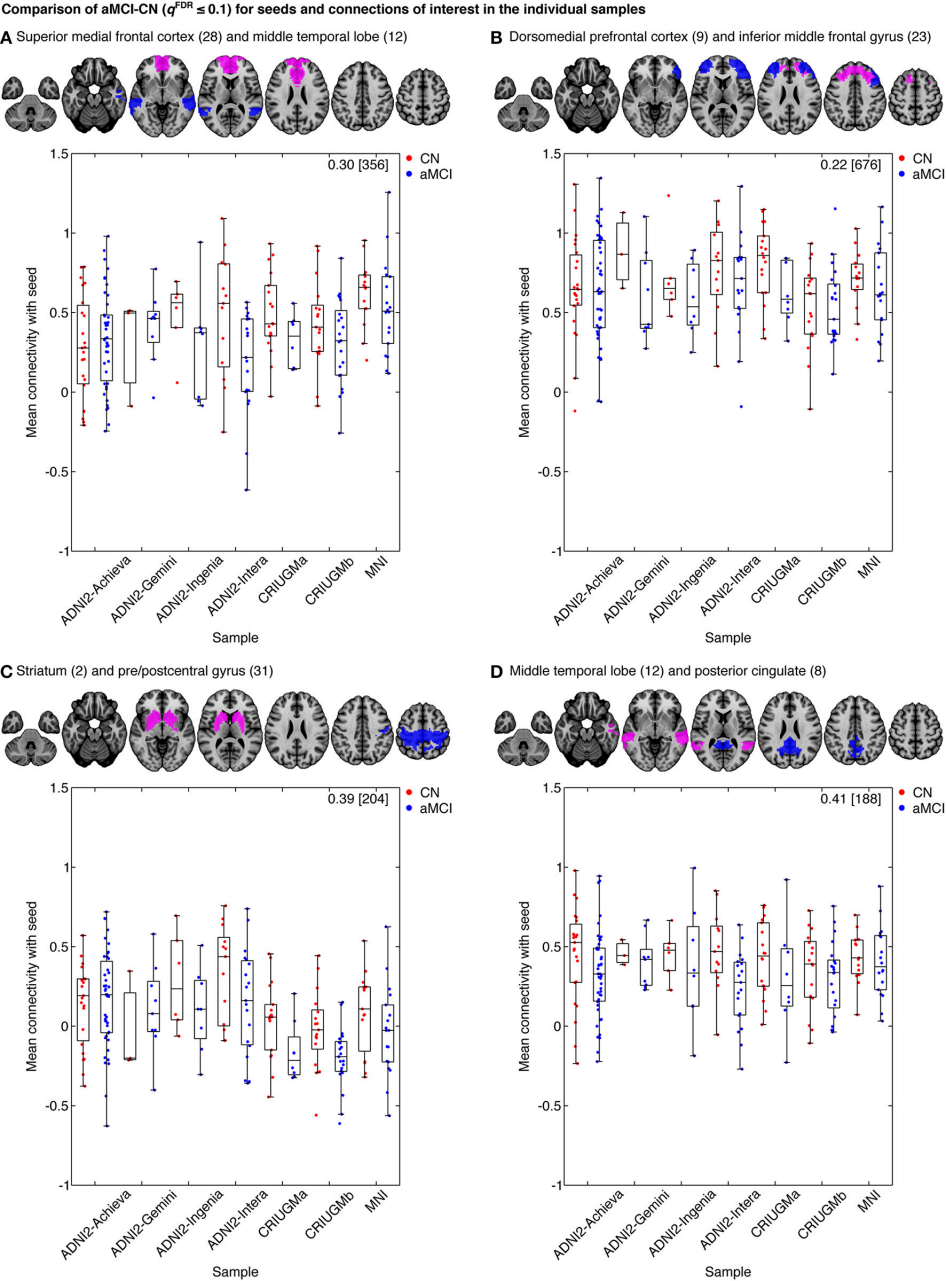
**Mean connectivity between (A) the superior medial frontal cortex and middle temporal lobe, (B) the dorsomedial prefrontal cortex and middle frontal gyrus, (C) the striatum and pre/postcentral gyrus, and (D) middle temporal lobe and posterior cingulate in CN and aMCI in the independent samples**. Each map displays the seed (pink) and a selected cluster (blue) whose connectivity with the seed significantly differed between CN and aMCI in the pooled analysis. The box-whisker plots display the mean connectivity (Fisher-transformed correlation values) between the seed and the selected parcel, overlaid over individual data points, in the CN and MCI groups in the ADNI2-Achieva, ADNI2-Gemini, ADNI2-Ingenia, ADNI2-Intera, CRIUGMa, CRIUGMb, and MNI samples. We also report the Cohen's *d* (a weighted average of the effect sizes per sample) followed by a sample size estimate (for 80% power, balanced groups, bilateral tests, Gaussian distributions, and α = 0.05) in square brackets in the top-right corner of each plot. The numbers in parentheses in the titles refer to the numerical IDs of the seeds in the 3D parcellation volume, as listed in Supplementary Table 2. For box-whisker plots for all significant clusters with each of these seeds, see Supplementary Figures 6–9.

However, despite the large observed variations in the spatial distribution of aMCI vs. CN contrasts, there were still clear consistent trends across studies and protocols. We indeed found that aMCI-related connectivity changes that surpassed the FDR threshold in the pooled analysis showed similar trends in the vast majority of samples across seeds and connections, where the independent aMCI samples consistently exhibited decreased connectivity compared to the CN samples (Figures 5, 6, Supplementary Figures 3–5). For example, the pooled analysis revealed that, compared to CN, aMCI exhibited significantly reduced connectivity between the superior medial frontal cortex cortex (the region in which connectivity was most affected by aMCI) and the middle temporal lobes. This change appeared to be common to the majority of the independent samples (Figures 5, 6A). For this particular seed, the change in connectivity was mainly due to regions with positive correlations in CN having smaller correlation values closer to zero in aMCI in the individual samples (Figures 5, 6A). For sample-specific effects in other seeds and connections, please see Supplementary Figures 3–9.

### Effect sizes and sample size estimates

We measured the effect sizes of the difference between groups at each significant connection by calculating Cohen's *d*, via a weighted average of the effect sizes per individual sample. We found small to medium effect sizes, ranging from *d* = 0.10–0.48, with an average effect size of *d* = 0.32. Note that these effect sizes are potentially inflated since we have focussed on significant results only. We also calculated the sample sizes required to achieve 80% power, based on the effect sizes estimated by Cohen's *d*, the assumption of balanced groups, Gaussian distributions, bilateral tests, and α = 0.05, for each connection. We found that the estimated sample sizes ranged from 140 to upwards of 600 total subjects, which further suggests that findings from small samples, similar to the seven samples we included when assessed independently, are not expected to be reliable. As noted above, as we used the same sample to estimate the location of effects and their size, these sample size estimates are possibly optimistic, i.e., deflated compared to a replication on an independent sample. See Figure 6 and Supplementary Figures 6–9 for Cohen's *d* and sample size estimates for each significant connection that was reported in Figure 4.

### Effect of resolution on the GLM

The percentage of discoveries in significant differences between aMCI and CN across the connectomes varied markedly as a function of resolution, as selected by the MSTEPS procedure. Higher resolutions were associated with fewer discoveries, especially beyond resolution 65 (Supplementary Figure 10A). By contrast, the maximal amplitude of differences in average connectivity associated with a particular cluster did not decrease substantially, and sometimes increased, when the resolution increased (Supplementary Figure 10B). The decrease in percentage of discovery thus likely reflected a cost associated with an increased number of multiple comparisons in the FDR procedure, rather than a loss in signal quality. Regarding the clusters that were selected for our seed-based analyses (the superior medial frontal cortex, dorsomedial prefrontal cortex, striatum, and middle temporal lobe), the associated effect maps (without statistical threshold) were highly consistent across different resolutions (Supplementary Figures 11, 12), with the potential exception of very low resolutions where, for example, a relatively small cluster like the anterior cingulate got merged with a large distributed cortical network. This also replicated a prior study on the effect of multiresolution parcellations on GLM analysis (Bellec et al., 2015). Lastly, signal-to-noise ratio did not have a significant impact on the results (Supplementary Figure 13).

## Discussion

We report resting-state functional connectivity differences in the superior medial frontal cortex, dorsomedial prefrontal cortex, striatum, and middle temporal lobe between aMCI and CN subjects when multiple studies were combined together. Despite protocol differences, we found that aMCI exhibited reduced connectivity within areas of the DMN and cortico-striatal-thalamic loop compared to CN. Previous studies suggested these altered patterns of functional connectivity in MCI may result from the coevolution of multiple AD-associated biological processes, namely structural degeneration (Pievani et al., 2010; Coupé et al., 2012), neurofibrillary and amyloid pathologies (Small et al., 2006), and cerebrovascular dysfunction (Villeneuve and Jagust, 2015).

The superior medial frontal cortex and middle temporal lobes, both of which are DMN nodes, were among the seed regions with the greatest amount of aMCI-related connectivity changes with other brain areas. Decreased connectivity in aMCI patients was found between these two nodes and other DMN regions, including the posterior cingulate, precuneus, inferior parietal lobes, ventromedial prefrontal cortex, and hippocampus. Our findings support previous studies that used small single-site samples and reported reduced DMN connectivity in MCI and AD patients (Sorg et al., 2007; Bai et al., 2009; Agosta et al., 2012; Koch et al., 2012). Alterations in the DMN may reflect increased amyloid burden in aMCI patients as it has been shown that amyloid plaques impair default mode connectivity (Hedden et al., 2009; Sheline et al., 2010b; Mormino et al., 2011).

We found reduced connectivity within the frontal lobes, notably between ventral and dorsal areas. Decreased functional connectivity between the ventral and dorsal frontal regions could reflect degeneration in gray matter and in white matter tracts connecting these areas. Longitudinal studies have shown greater prefrontal cortex atrophy in MCI over time, as well as in those transitioning to AD, compared to CN (McDonald et al., 2009; Carmichael et al., 2013). Cortico-cortical white matter bundles, e.g., superior longitudinal fasciculus, have also been demonstrated to degenerate in patients with MCI and AD (Pievani et al., 2010). Additionally, functional connectivity changes may reflect the regional effect of increased amyloid burden (Sheline et al., 2010b), and PIB-PET work has shown the frontal lobe to be one of the first regions in which amyloid accumulates in autosomal dominant AD mutation carriers (Bateman et al., 2012). Our results may also be due to neurofibrillary pathology as it typically appears in the prefrontal cortex during MCI (Bossers et al., 2010). Lastly, cerebral hypoperfusion in the frontal lobe of MCI (Chao et al., 2009) may have contributed to our results.

We also observed functional disconnection between the temporal and frontal lobes in aMCI. Effects in the temporal lobes were expected given that the temporal lobe is a region known to suffer from significant AD pathology in preclinical phases (Guillozet et al., 2003). Structural connectivity may also explain the functional connectivity changes between the frontal and temporal regions, since degeneration of white matter tracts between these areas, e.g., the uncinate fasciculus, occurs with the progression from MCI to AD and correlates with episodic memory impairment in MCI (Pievani et al., 2010; Rémy et al., 2015). Furthermore, examining the integrity of the arcuate fasciculus, a major language tract that connects the frontal and temporal lobes (Dick and Tremblay, 2012), might reveal a biological basis for language impairments such as word-finding difficulties in MCI and AD, (Nutter-Upham et al., 2008). Brain areas that subserve language function could be important targets to investigate given recent evidence that multilingualism, like other forms of cognitive reserve, may help delay the onset of AD (Chertkow et al., 2010).

Unexpectedly, we also found significant effects in the striatum, which showed reduced connectivity in aMCI with the sensorimotor cortex, frontal and parietal regions, and thalamus. While not initially expected, these findings may reflect earlier observations that regions within the cortico-striatal-thalamic loops are vulnerable to AD pathology. For example, previous work demonstrated the presence of substantial amyloid burden in the striatum in both autosomal dominant and sporadic forms of AD (Braak and Braak, 1990; Villemagne et al., 2009), and the striatum may be the first region in which amyloid deposition occurs in autosomal dominant AD (Klunk et al., 2007; Bateman et al., 2012). Furthermore, significant neurodegeneration is known to occur with AD in the striatum and thalamus (de Jong et al., 2008; Madsen et al., 2010), so our results might reflect the brain's capacity for functional plasticity in response to amyloid or neurodegeneration in these regions. Motor cortex hyperexcitability has also been shown in AD, and this suggests that inhibitory circuits leading to the motor cortex may be affected in the disease (Ferreri et al., 2011). Patients with AD also demonstrate changes in swallowing which have been associated with altered cortical activity (Humbert et al., 2010). Our results may support these observations. Additionally, our findings may represent a biological basis for the cognitive and motor symptoms of MCI (Aggarwal et al., 2006) since the striatum and the rest of the basal ganglia have been implicated in stimulus-response associative learning and memory and motor skill acquisition and execution (Packard and Knowlton, 2002; Doyon et al., 2009). Future research should examine the potential relationship between connectivity in the cortico-striatal-thalamic loops and motor function in aMCI and AD.

Our findings contrasted with previous, smaller single-site studies that have variously reported decreased and increased connectivity. The reports of increased connectivity (Bai et al., 2009; Qi et al., 2010; Gour et al., 2011) may have reflected unique attributes of particular protocols or the choices made with respect to pre-processing steps, for example using global signal regression (Saad et al., 2012). Given that our sample size estimates suggest the use of hundreds of subjects to obtain adequate statistical power, it is not surprising that discrepancies between our results and previous findings generated from smaller, likely underpowered, studies exist. Even when we examined the samples in our study (ADNI2-Achieva, ADNI2-Gemini, ADNI2-Ingenia, ADNI2-Intera, CRIUGMa, CRIUGMb, MNI) independently of each other, we found inconsistent effects among the samples. It is only by combining the studies together in a meta-analysis that we were able to find some common differences in functional connectomes between patients with aMCI and CN. This finding underscores the need for multisite studies with large sample sizes in order to generate reproducible results, as previously suggested in the field of autism research (Haar et al., 2014).

Among our study's limitations is that it was not possible to model each of the 13 ADNI2 sites independently because the sites tended to be small and unbalanced in the numbers of patients and controls. We therefore chose to model each scanner model within ADNI2 separately based on the recommendation of a reviewer. A previous version of the analysis (published as a preprint^9^) had not modeled the different scanner models in ADNI2 and instead treated ADNI2 as a single site. This previous analysis yielded fewer significant findings, but the results were still mostly consistent with what is reported here. Our results suggest that modeling scanner models may have a positive impact on fMRI association studies, but further experiments would be required to confirm that this trend is reproducible. We must also note that the METAL averaging is only representative of the specific samples that were averaged, especially using only Philips and Siemens scanners, and it is unclear how our findings may replicate in other studies that would employ a different combination of protocols, say using GE scanners. In particular, our sample size estimates have to be interpreted with caution. They may first be under-estimated, because they were not derived from pre-specified locations, but rather associated with the connections showing the largest effects in our particular sample. These sample sizes were also derived from a meta-analysis combining particular types of studies. We only had 3T scanners from two manufacturers, Siemens and Philips. For the Siemens studies, all were from the same model. For the Philips studies, the scanning protocol was identical at every site, and only the scanner model varied across scanners. Finally, a fairly large number of patients and controls (generally more than 10 subjects per group) was scanned for each variant of the scanning protocol. The sample size estimate may turn out quite differently for a single site study or on the contrary for a study with a very large number of sites and with only a few subjects per site.

Our study is also limited by its cross-sectional nature, which precludes inference that the functional changes we found would necessarily predict progression toward Alzheimer's dementia. Furthermore, aMCI has many underlying causes aside from AD. It is possible that some subjects in our cohort had cognitive impairments due to Lewy Body dementia, for example. However, all samples in the current study had inclusion criteria that enriched for subjects that had aMCI likely due to AD and excluded aMCI subjects with other co-morbidities, such as depression or Parkinson's disease. Also, we did not account for structural atrophy, despite a bias for increased detection in functional differences due to differences in underlying structure (Dukart and Bertolino, 2014). However, aMCI-related gray matter changes likely co-localize to some extent with functional changes, and the aim of our work was to map out functional changes rather than study their interaction with atrophy. We did not account for other variables, such as APOE genotype (Sheline et al., 2010a), amyloid deposition (Sheline et al., 2010b), presence of neurofibrillary tangles (Maruyama et al., 2013), and cerebrovascular mechanisms (Villeneuve and Jagust, 2015). At least some of these could potentially have explained the observed aMCI-related functional connectivity changes as part of an underlying disease mechanism. Large-scale multimodal studies, incorporating genomics, proteomics, and multimodal imaging will be needed to identify the interactions between these and other physiological facets of the pathology. Despite combining several samples together, we still only achieved relatively limited power, given that sample size estimates required at least 140 to over 600 total subjects to consistently identify effects between groups. Lastly, because of the explorative approach used in our study, the resulting estimates of effect sizes may have been inflated and discussion of possible pathological mechanisms for our findings was speculative. However, our discoveries may be used as follow-up targets in future work. Upcoming research should not only attempt to verify our findings by using these regions and their associated connections with hypothesis-driven approaches (e.g., seed-based correlation analyses), but also to extend them to cohorts that include Alzheimer's dementia and other clinical populations (e.g., CN with significant amyloid deposition) and to longitudinal studies that characterize individuals' progression to dementia. Finally, future studies should aim to determine whether our findings are associated with established biomarkers of AD (e.g., amyloid and tau quantification) in order to probe the potential of these functional connections as biomarkers.

Overall, our results supported previous findings of DMN connectivity changes in AD and MCI (Greicius et al., 2004; Sorg et al., 2007), given that three of the identified seeds (superior medial frontal cortex, dorsomedial prefrontal cortex, middle temporal lobe) are part of this network. It is noteworthy, however, that our strongest observed effects reported here were not in the same DMN regions typically described in earlier resting-state studies of MCI and AD, viz, posterior cingulate/precuneus (Sheline et al., 2010b; Zhang et al., 2010). Unexpected changes were also found in the striatum, and this may reflect the advantages of “mining” the whole-brain connectome to search for new biomarkers of mild cognitive impairment and possibly the early progression of the pathophysiologic substrate of Alzheimer's disease. If confirmed, our results could suggest the utility of these regions in resting-state fMRI as a biomarker endpoint in clinical trials.

### Conflict of interest statement

The authors declare that the research was conducted in the absence of any commercial or financial relationships that could be construed as a potential conflict of interest.

## Footnotes

^1^http://simexp.github.io/niak/.

^2^https://www.gnu.org/software/octave/.

^3^http://www.bic.mni.mcgill.ca/ServicesSoftware/ServicesSoftwareMincToolKit.

^4^http://www.calculquebec.ca/en/resources/compute-servers/guillimin.

^5^https://github.com/SIMEXP/mcinet.

^6^http://niak.simexp-lab.org/pipe_preprocessing.html.

^7^https://github.com/SIMEXP/mcinet/tree/master/preprocess.

^8^http://dx.doi.org/10.6084/m9.figshare.1480461.

^9^http://dx.doi.org/10.1101/019646.
