# Supplementary material for: Common Effects of Amnestic Mild Cognitive Impairment on Resting-State Connectivity Across Four Independent Studies
Source: Front Aging Neurosci. 2015 Dec 24;7:242. doi: 10.3389/fnagi.2015.00242 (PMC4689788; doi:10.3389/fnagi.2015.00242)
Supplement: Supplementary file 1 [file Image1.PDF]

Default mode network subcomponents across resolutions

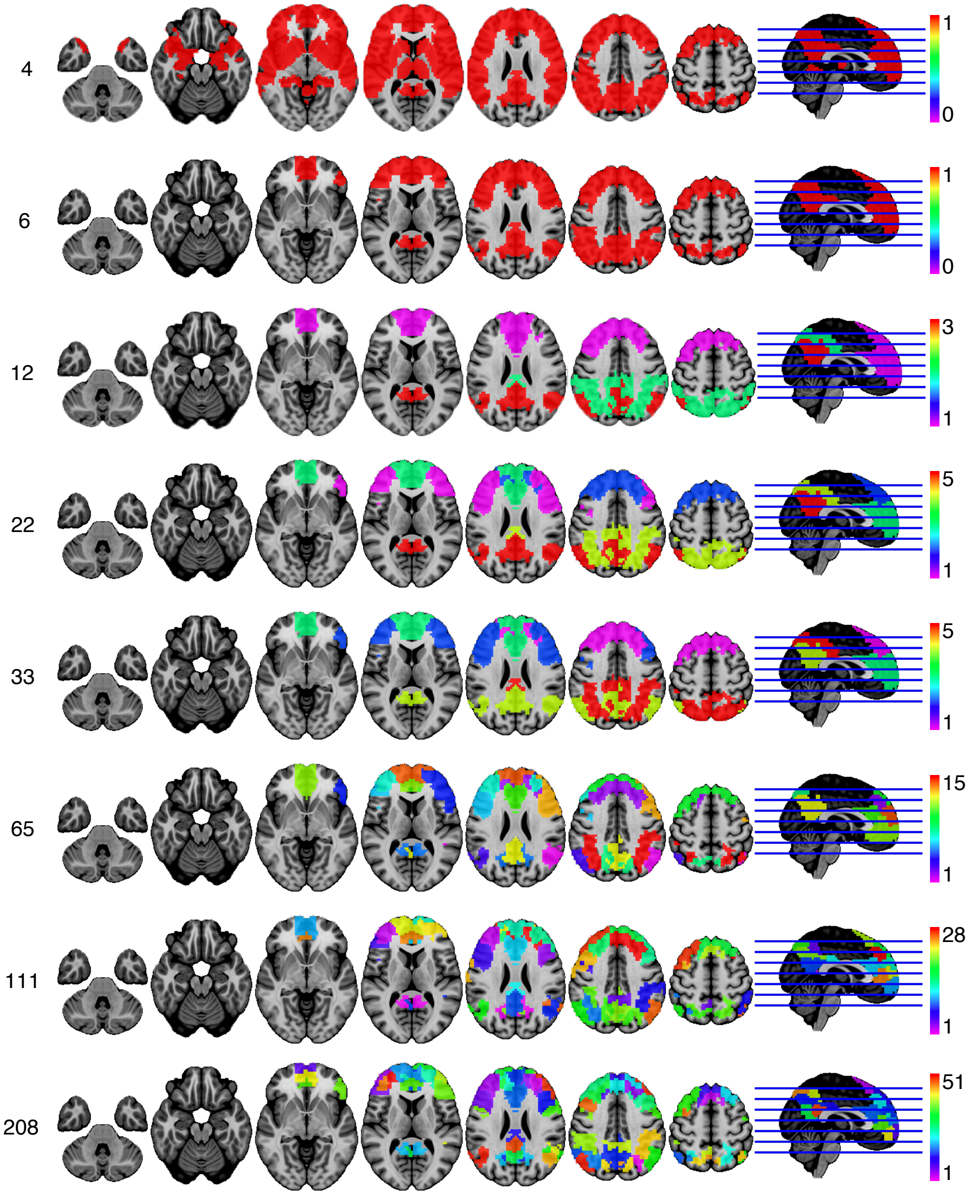

Supplementary Figure 1. The default mode network and its subcomponents across resolutions (or number of clusters) selected by MSTEPS.
