# Supplementary material for: Common Effects of Amnestic Mild Cognitive Impairment on Resting-State Connectivity Across Four Independent Studies
Source: Front Aging Neurosci. 2015 Dec 24;7:242. doi: 10.3389/fnagi.2015.00242 (PMC4689788; doi:10.3389/fnagi.2015.00242)
Supplement: Supplementary file 3 [file Image3.PDF]

Comparisons of effects across samples

Dorsomedial prefrontal cortex (9)

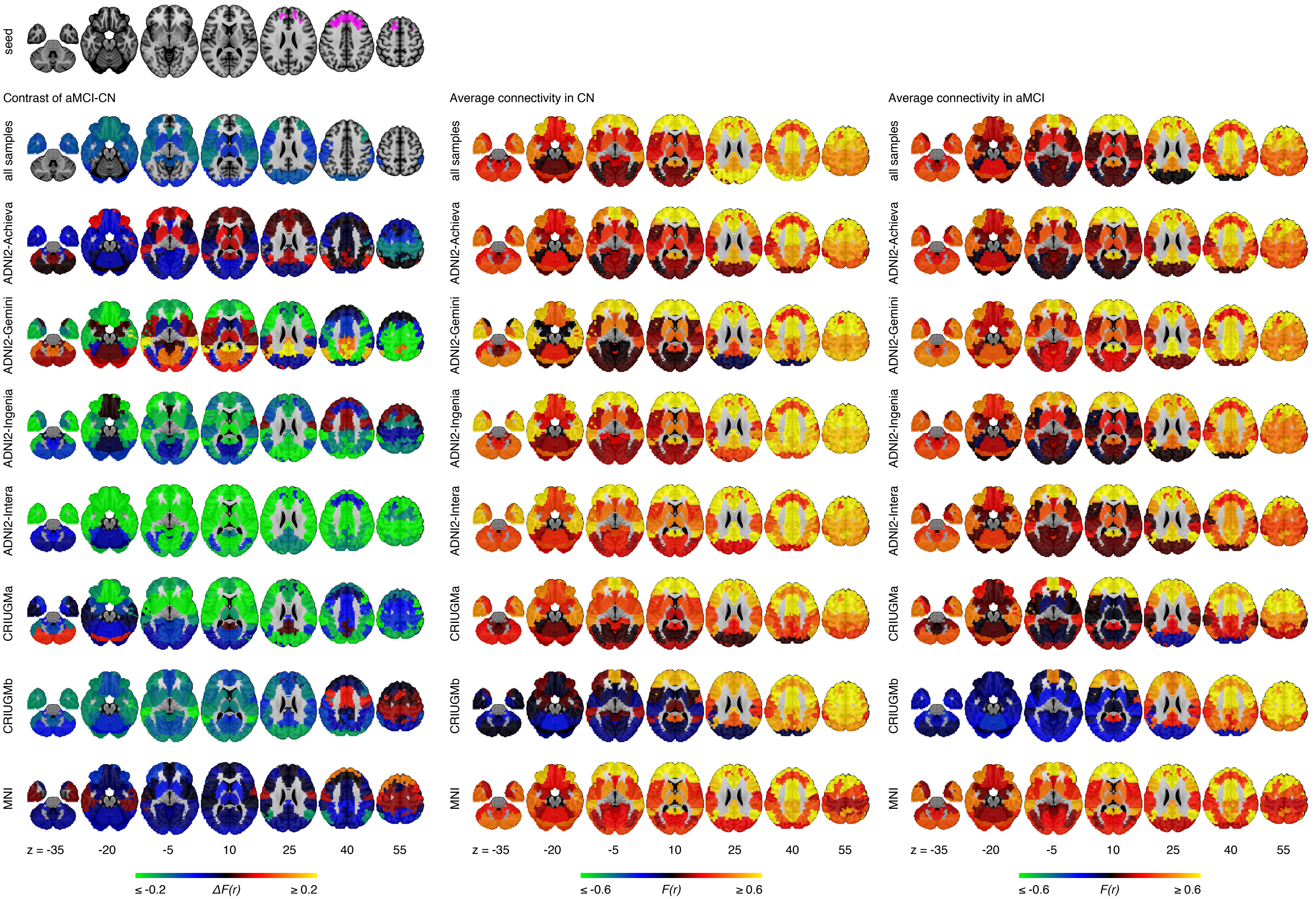

Supplementary Figure 3. Comparisons of effects in the dorsomedial prefrontal cortex across samples. This figure illustrates functional connectivity changes between aMCI and CN, average connectivity in CN, and average connectivity in aMCI in each site (ADNI2-Achieva, ADNI2-Gemini, ADNI2-Ingenia, ADNI2-Intera, CRIUGMa, CRIUGMb, MNI) independently of other sites and when samples are pooled together (all samples). The number in parentheses refers to the numerical ID of the seed in the 3D parcellation volume, as listed in Supplementary Table 2.
