# Supplementary material for: Common Effects of Amnestic Mild Cognitive Impairment on Resting-State Connectivity Across Four Independent Studies
Source: Front Aging Neurosci. 2015 Dec 24;7:242. doi: 10.3389/fnagi.2015.00242 (PMC4689788; doi:10.3389/fnagi.2015.00242)
Supplement: Supplementary file 8 [file Image8.PDF]

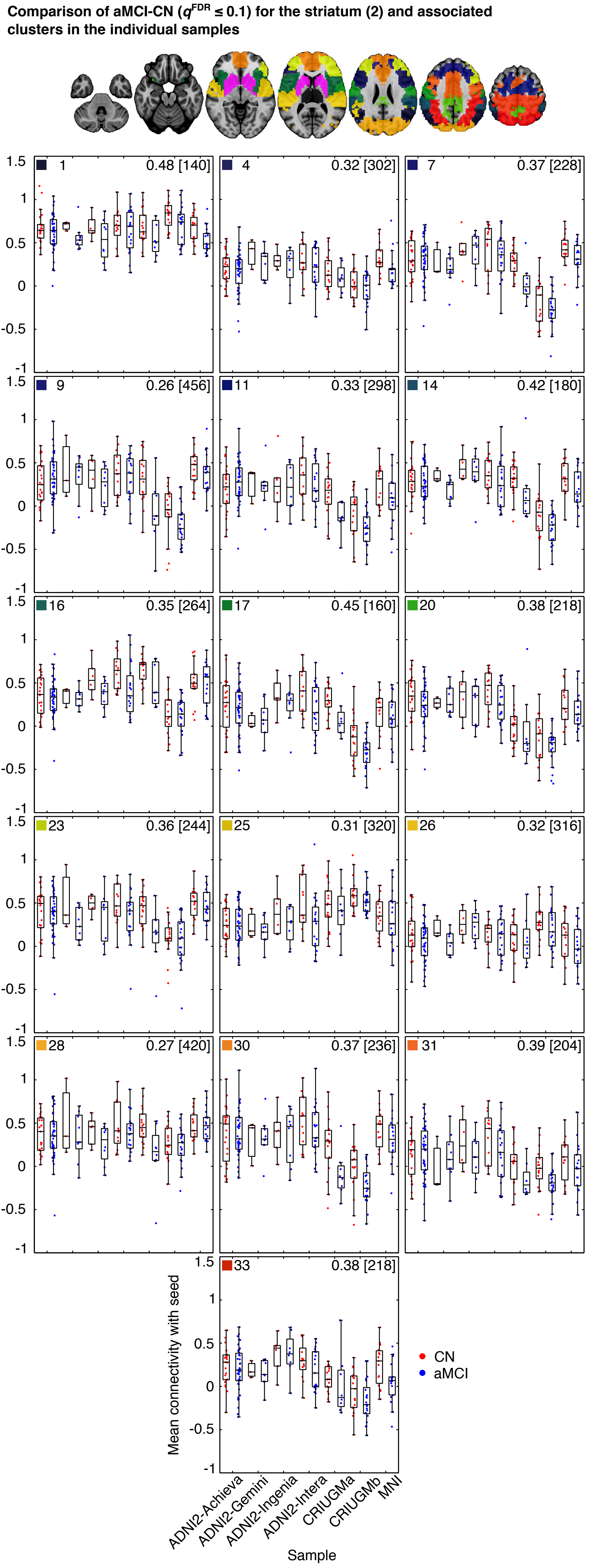

Supplementary Figure 8. Mean connectivity of the striatum (cluster #2) with its associated connections in CN and aMCI in the independent samples. Each map displays the seed in pink and the clusters (in other colors) whose connectivity with the seed significantly differed between CN and aMCI in the pooled analysis. The box-whisker plots display the mean connectivity (Fisher-transformed correlation values) between the seed and a significant parcel, overlaid over individual data points, in the CN and aMCI groups in the ADNI2-Achieva, ADNI2-Gemini, ADNI2-Ingenia, ADNI2-Intera, CRIUGMa, CRIUGMb, and MNI samples. Each plot is labeled with a number in the top-left corner, corresponding to the number assigned to the cluster in Supplementary Table 2, and a colored square corresponding to the parcel of the same color from the map. We also report the Cohen's d (a weighted average of the effect sizes per sample) followed by a sample size estimate (for 80% power, balanced groups, bilateral tests, Gaussian distributions, and $\alpha = 0.05$) in square brackets in the top-right corner of each plot.
