# Supplementary material for: Common Effects of Amnestic Mild Cognitive Impairment on Resting-State Connectivity Across Four Independent Studies
Source: Front Aging Neurosci. 2015 Dec 24;7:242. doi: 10.3389/fnagi.2015.00242 (PMC4689788; doi:10.3389/fnagi.2015.00242)
Supplement: Supplementary file 10 [file Image10.PDF]

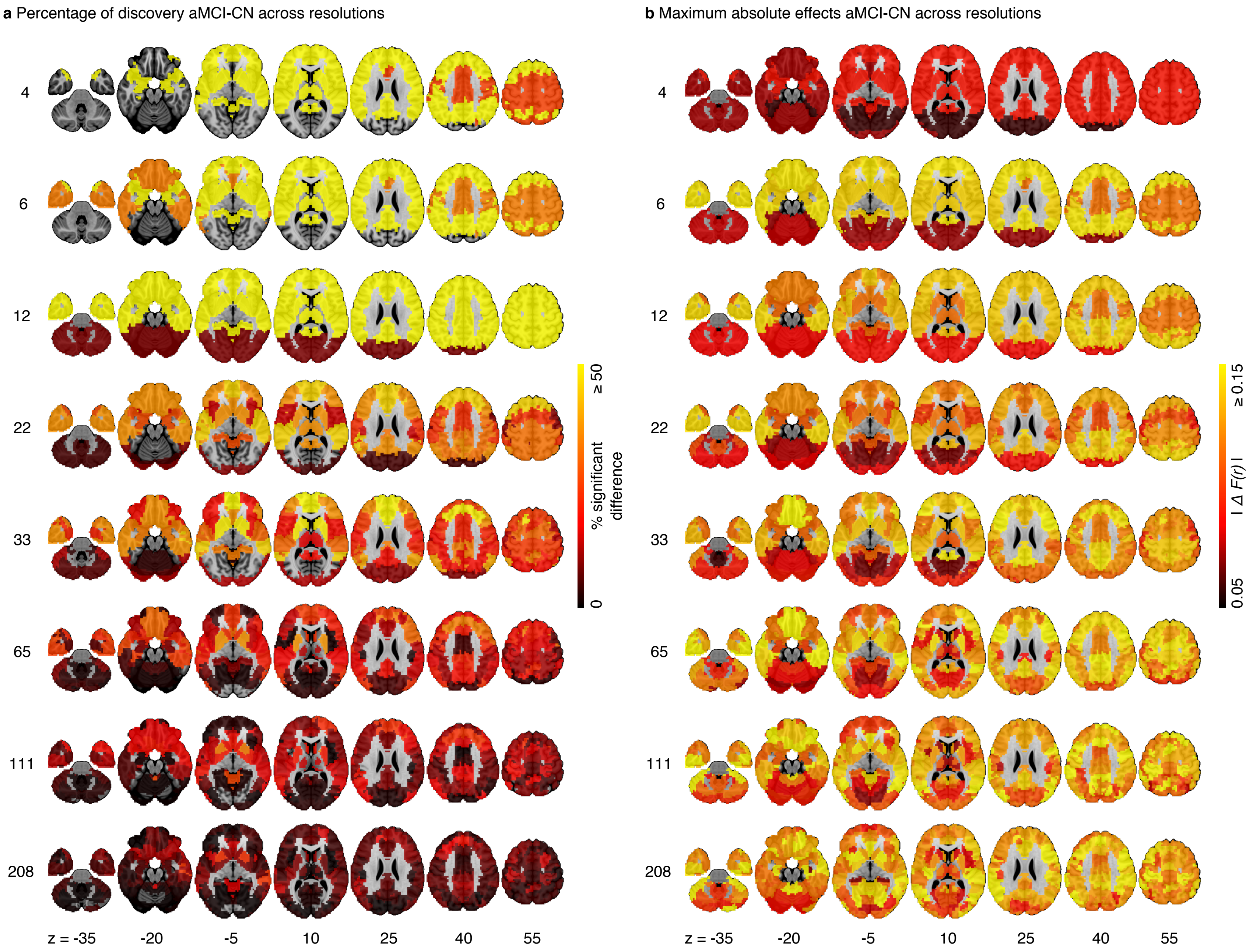

Supplementary Figure 10. Comparison of results across different resolutions (or number of clusters) selected by MSTEPS. a) Maps of percentage of discovery illustrating brain networks that are significantly different between aMCI and CN. b) Maps of maximum absolute effects show the magnitude of the differences between aMCI and CN of every cluster.
