# Supplementary material for: Common Effects of Amnestic Mild Cognitive Impairment on Resting-State Connectivity Across Four Independent Studies
Source: Front Aging Neurosci. 2015 Dec 24;7:242. doi: 10.3389/fnagi.2015.00242 (PMC4689788; doi:10.3389/fnagi.2015.00242)
Supplement: Supplementary file 11 [file Image11.PDF]

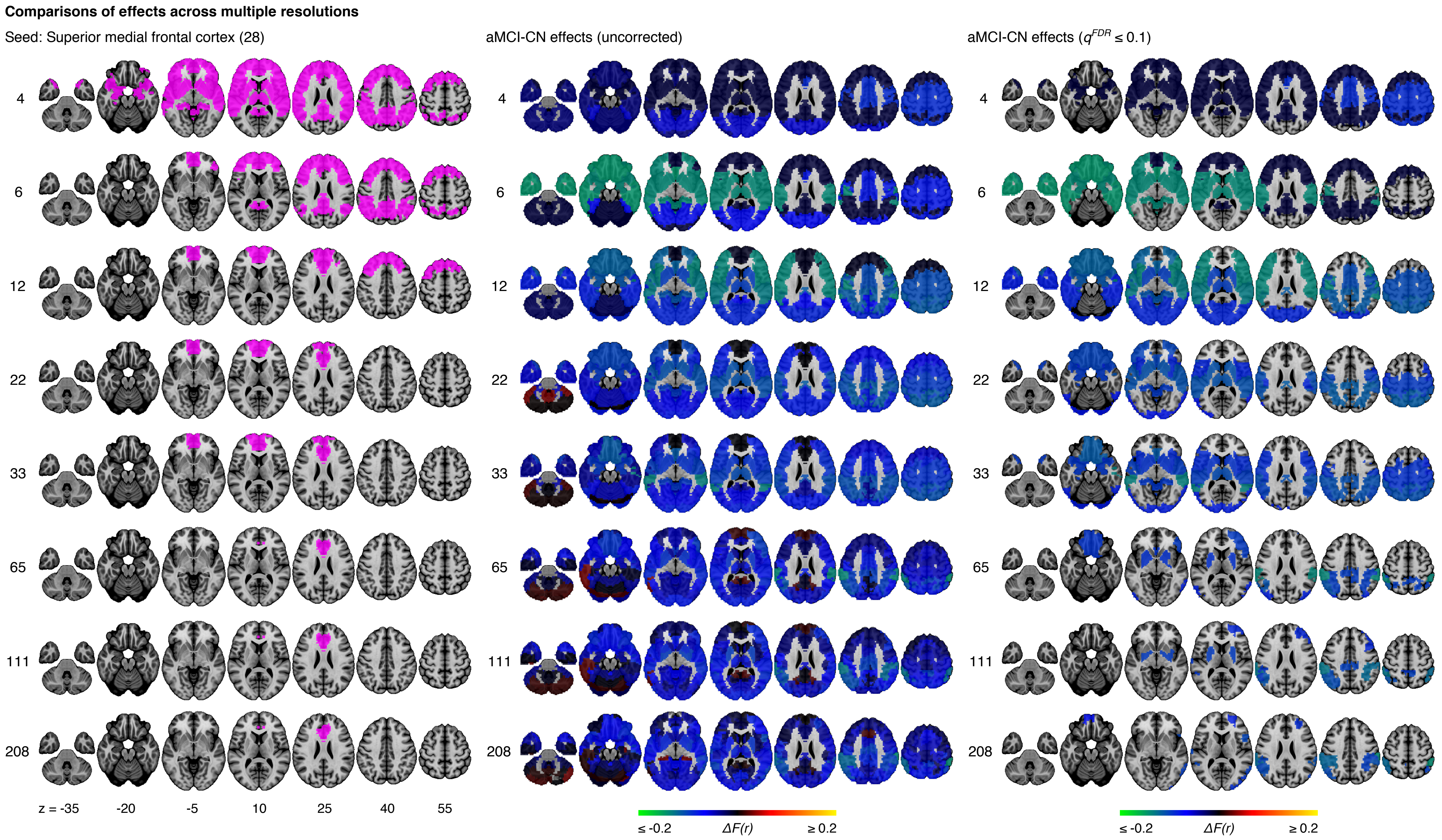


**Supplementary Figure 11.** Comparisons of uncorrected and FDR-corrected effects related to aMCI across multiple resolutions selected by MSTEPS in the superior medial frontal cortex as the seed. The number in parentheses refers to the numerical ID of the seed in the 3D parcellation volume for 33 clusters, as listed in Supplementary Table 2.
