# Supplementary figures and images for: Common Effects of Amnestic Mild Cognitive Impairment on Resting-State Connectivity Across Four Independent Studies
Source: Front Aging Neurosci. 2015 Dec 24;7:242. doi: 10.3389/fnagi.2015.00242 (PMC4689788; doi:10.3389/fnagi.2015.00242)

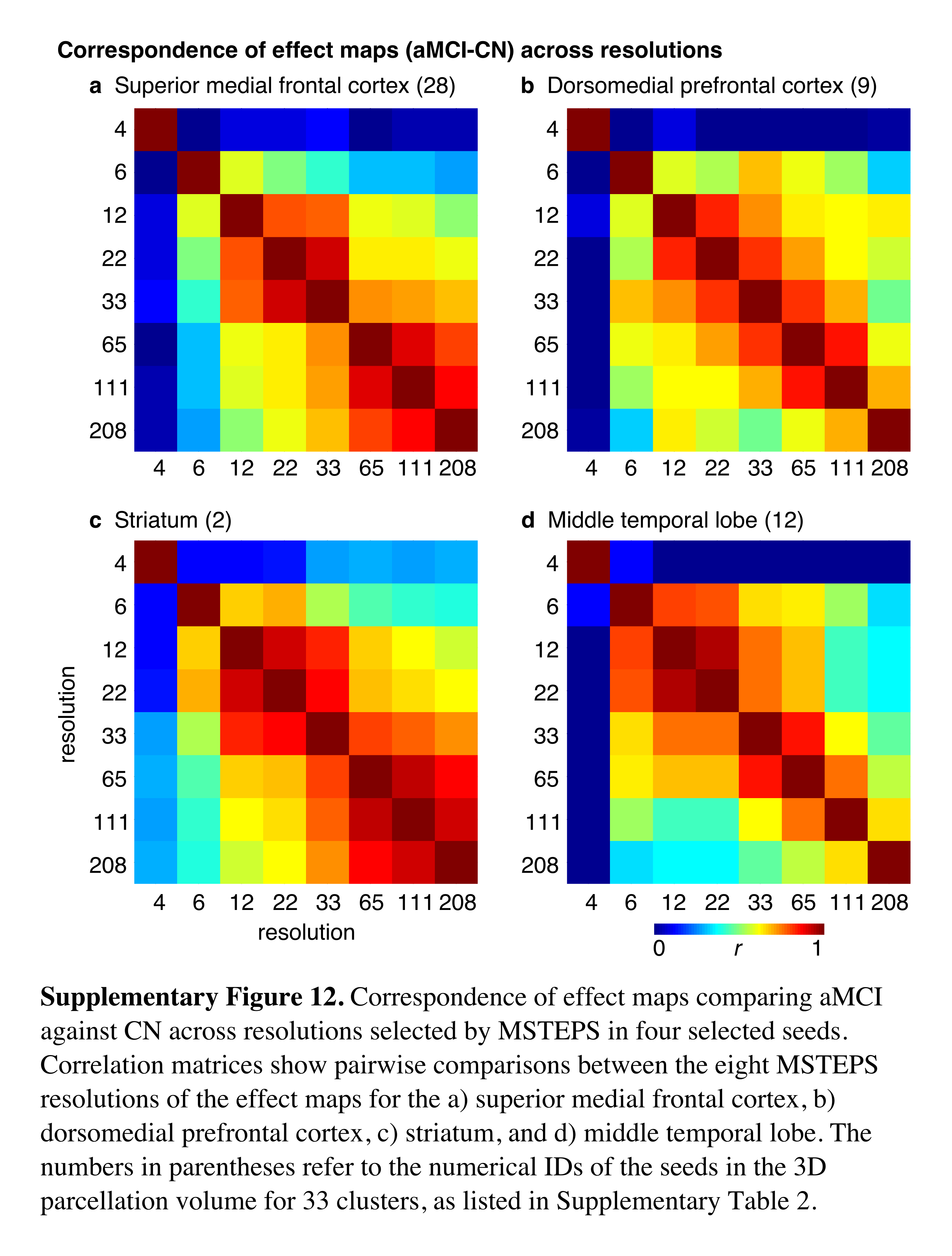

Supplement: Supplementary file 12 [file Image12.png]
