# Supplementary material for: Common Effects of Amnestic Mild Cognitive Impairment on Resting-State Connectivity Across Four Independent Studies
Source: Front Aging Neurosci. 2015 Dec 24;7:242. doi: 10.3389/fnagi.2015.00242 (PMC4689788; doi:10.3389/fnagi.2015.00242)
Supplement: Supplementary file 13 [file Image13.PDF]

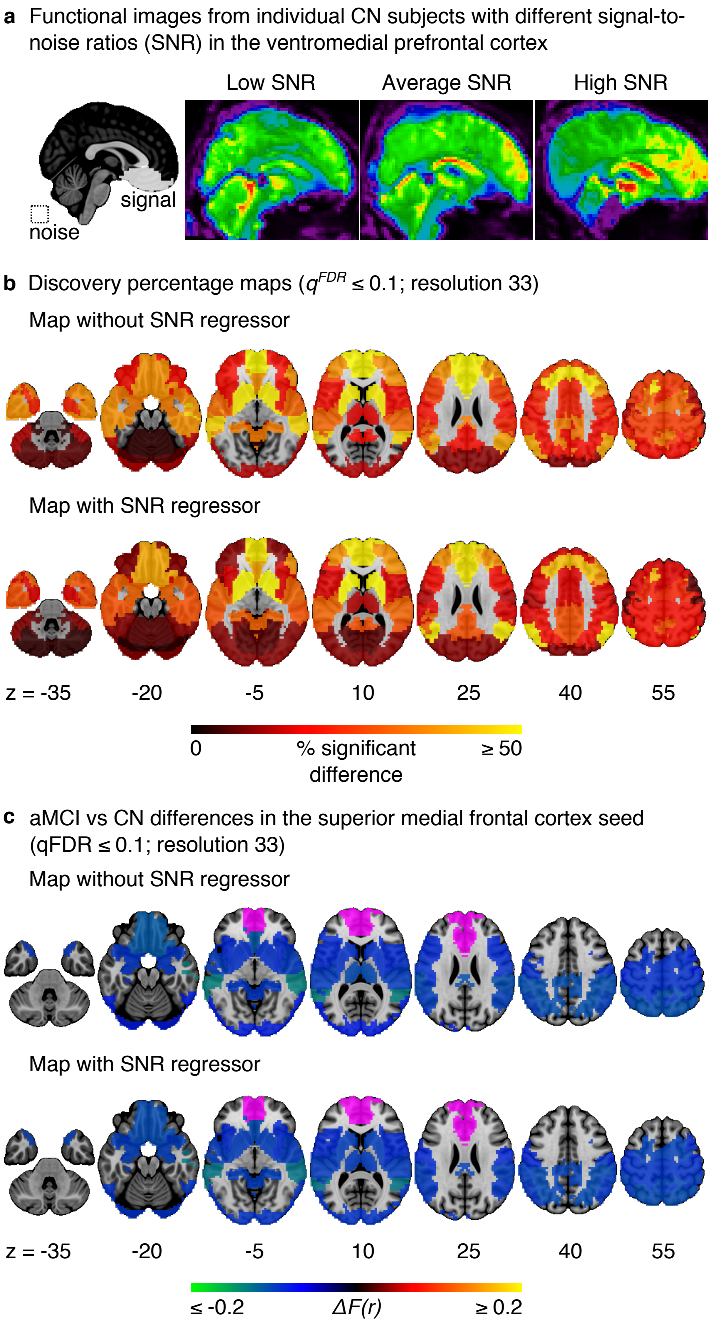

Supplementary Figure 13. Examination of signal-to-noise ratio (SNR) in the ventromedial prefrontal cortex on the results from the general linear model (GLM). a) For each individual, we calculated the signal in the ventromedial prefrontal cortex (VMPFC) (displayed in white) and the noise, defined as the standard deviation of values in a small square outside the brain. The SNR was the VMPFC signal divided by the standard deviation of the square. Here, we present individuals with low, average, and high SNR. b) Maps of the percentage of connections associated with a given cluster and identified as significant by the statistical comparison between aMCI and CN, at a resolution of 33 clusters (qFDR ≤ 0.1) in the original GLM (without SNR regressor) and the new GLM with the SNR regressor. c) Difference maps between aMCI and CN for the functional connectivity between the superior medial frontal cortex (pink) and the rest of the brain. ΔF(r) denotes difference in Fisher-transformed correlation values. All connections shown in the maps of difference in average connectivity between aMCI and CN are significant at qFDR ≤ 0.1. Top line is the original GLM and the bottom line is the new GLM with the SNR regressor.
