# Supplementary material for: Common Effects of Amnestic Mild Cognitive Impairment on Resting-State Connectivity Across Four Independent Studies
Source: Front Aging Neurosci. 2015 Dec 24;7:242. doi: 10.3389/fnagi.2015.00242 (PMC4689788; doi:10.3389/fnagi.2015.00242)
Supplement: Supplementary file 14 [file Table1.DOCX]

Supplementary Table 1. Demographic information in all studies before quality control

|  |  | **ADNI2** | **CRIUGMa** | **CRIUGMb** | **MNI** | **Combined**  **sample** |
| --- | --- | --- | --- | --- | --- | --- |
| **CN** | N | 59 | 20 | 18 | 15 | 112 |
|  | Mean age (s.d.) | 74.1 (6.8) | 71.7 (8.2) | 69.9 (4.9) | 67.0 (5.7) | 72.0 (7.0) |
|  | Number male (%) | 27 (46%) | 7 (35%) | 2 (11%) | 7 (47%) | 43 (38%) |
|  | MMSE mean (range) | 28.8 (24-30) | 28.8 (27-30) | n/a | 29.1 (27-30) | n/a |
|  | MoCA mean (range) | n/a | 27.6 (22-30) | 28.5 (26-30) | n/a | n/a |
|  |  |  |  |  |  |  |
| **aMCI** | N | 93 | 9 | 22 | 19 | 143 |
|  | Mean age (s.d.) | 71.7 (7.8) | 80.7 (6.2) | 74.9 (6.9) | 71.6 (8.1) | 72.7 (7.7) |
|  | Number male (%) | 47 (51%) | 4 (44%) | 13 (59%) | 8 (42%) | 72 (50%) |
|  | MMSE mean (range) | 28.1 (24-30) | 26.3 (22-29) | n/a | 25.9 (22-30) | n/a |
|  | MoCA mean (range) | n/a | 23.6 (20-29) | 25.0 (16-29) | n/a | n/a |
