# Supplementary material for: Common Effects of Amnestic Mild Cognitive Impairment on Resting-State Connectivity Across Four Independent Studies
Source: Front Aging Neurosci. 2015 Dec 24;7:242. doi: 10.3389/fnagi.2015.00242 (PMC4689788; doi:10.3389/fnagi.2015.00242)
Supplement: Supplementary file 15 [file Table2.DOCX]

Supplementary Table 2. Numerical IDs, labels, and volumes (mm^3^) of parcels in the symmetric and asymmetric brain parcellations containing 33 clusters

| ID | Label | Volume (Symmetric) | Volume (Asymmetric) |
| --- | --- | --- | --- |
| 1 | Thalamus | 20547 | 20547 |
| 2 | Striatum | 47574 | 47574 |
| 3 | Cerebellum_8_9_10 | 25461 | 25461 |
| 4 | Angular/Parietal_Inf | 34587 | 34074 |
| 5 | Temporal_Pole | 28242 | 28350 |
| 6 | Cerebellum_3_4_5_6 | 42822 | 42768 |
| 7 | Frontal_Inf_Tri | 31860 | 31671 |
| 8 | Cingulum_Post | 38934 | 39204 |
| 9 | Prefrontal_Dorsomedial | 36693 | 35748 |
| 10 | Hippocampus | 57024 | 56997 |
| 11 | Frontal_Sup | 30969 | 31239 |
| 12 | Temporal_Mid | 32184 | 32157 |
| 13 | Frontal_Mid/Sup | 38043 | 36909 |
| 14 | Supramarginal/Parietal_Inf | 32670 | 31185 |
| 15 | Cerebellum_Crus_Ant | 48762 | 48843 |
| 16 | Frontal_Inf_Oper | 58239 | 57888 |
| 17 | Postcentral | 29538 | 29403 |
| 18 | Precentral | 44496 | 44334 |
| 19 | Prefrontal_Ventrolateral | 58158 | 58077 |
| 20 | Precuneus | 30051 | 30024 |
| 21 | Fusiform/Parahippocampal | 83997 | 82917 |
| 22 | Prefrontal_Ventromedial | 46035 | 47493 |
| 23 | Frontal_Inf/Mid | 57240 | 58266 |
| 24 | Occipital_Mid/Inf | 58671 | 61020 |
| 25 | Temporal_Sup/Insula | 65205 | 63666 |
| 26 | Cuneus | 60669 | 67230 |
| 27 | Cerebellum_Crus_Post | 76086 | 76086 |
| 28 | Frontal_Sup_Medial | 62424 | 62262 |
| 29 | Calcarine/Lingual | 57564 | 57483 |
| 30 | Precentral/Supplementary_Motor | 54135 | 54891 |
| 31 | Pre/postcentral | 70173 | 69606 |
| 32 | Calcarine/Lingual/Cuneus | 77328 | 77382 |
| 33 | Occipital_Sup | 80919 | 80190 |
