# Supplementary material for: Common Effects of Amnestic Mild Cognitive Impairment on Resting-State Connectivity Across Four Independent Studies
Source: Front Aging Neurosci. 2015 Dec 24;7:242. doi: 10.3389/fnagi.2015.00242 (PMC4689788; doi:10.3389/fnagi.2015.00242)
Supplement: Supplementary file 16 [file Table3.DOCX]

Supplementary Table 3. Rank of parcels based on their associated percentage of non-redundant connections that differ between aMCI and CN

| **Rank** | **Parcel** | **Additional percentage of connections** | **Cumulative percentage of connections** |
| --- | --- | --- | --- |
| 1 | Superior medial frontal cortex (28) | 13.44 | 13.44 |
| 2 | Dorsomedial prefrontal cortex (9) | 12.65 | 26.09 |
| 3 | Striatum (2) | 11.06 | 37.15 |
| 4 | Middle temporal lobe (12) | 9.89 | 47.04 |
| 5 | Fusiform/Parahippocampal (21) | 8.69 | 55.73 |
| 6 | Angular/Inferior parietal (4) | 7.12 | 62.85 |
| 7 | Hippocampus (10) | 6.32 | 69.17 |
| 8 | Ventromedial prefrontal cortex (22) | 6.32 | 75.49 |
| 9 | Temporal pole (5) | 4.75 | 80.24 |
| 10 | Middle/Superior frontal cortex (13) | 4.74 | 84.98 |
| 11 | Superior temporal/Insula (25) | 4.74 | 89.72 |
| 12 | Inferior/Middle frontal cortex (23) | 3.17 | 92.89 |
| 13 | Prefrontal ventrolateral cortex (19) | 2.37 | 95.26 |
| 14 | Posterior cerebellar crus (27) | 1.58 | 96.84 |
| 15 | Calcarine/Lingual/Cuneus (32) | 1.58 | 98.42 |
| 16 | Anterior cerebellar crus (15) | 0.79 | 99.21 |
| 17 | Inferior frontal operculum (16) | 0.79 | 100.00 |

Numbers in parentheses reference the number assigned to the cluster as per Supplementary Table 2
