# Supplementary material for: Common Effects of Amnestic Mild Cognitive Impairment on Resting-State Connectivity Across Four Independent Studies
Source: Front Aging Neurosci. 2015 Dec 24;7:242. doi: 10.3389/fnagi.2015.00242 (PMC4689788; doi:10.3389/fnagi.2015.00242)
Supplement: Supplementary file 17 [file DataSheet1.DOCX]

Supplementary Methods

Inclusion/exclusion criteria by each individual study

ADNI2

Subjects must have been either English or Spanish-speaking and between 55-90 (inclusive) years of age. All subjects had a partner able to provide an independent evaluation of functioning. Criteria for CN subjects were as follows: MMSE scores between 24-30 (inclusive), a CDR of 0, non-depressed, non-MCI, and non-demented. Criteria for aMCI were as follows: MMSE scores between 24-30 (inclusive), a memory complaint, have objective memory loss measured by education adjusted scores on Wechsler Memory Scale Logical Memory II, a CDR of 0.5, absence of significant levels of impairment in other cognitive domains, essentially preserved activities of daily living, and an absence of dementia.

CRIUGMa

All subjects must have been 50 years of age or older, French-speaking or bilingual, and lived in Quebec for most of their lives. Exclusion criteria included history of psychiatric or neurological disorders, history of substance abuse, and having undergone anesthesia within the last six months of participating in the study. aMCI must have subjective memory complaints, an objective memory deficit (measured as a score at least 1.5 standard deviations below the mean considering age and education), and intact functional abilities. aMCI must not have met criteria for dementia. CN subjects must not have met criteria for MCI or dementia.

CRIUGMb

All subjects must have been between 65 and 80 years old, had normal audition and vision at the time of study, and at least 8 years of education. Exclusion criteria included family history of early-onset AD, use of psychoactive substances within last 3 months, intellectual disabilities, signs of depression. aMCI must have had memory complaints, an MMSE score equal to or greater than 24, a CDR of 0.5, and cognitive deficits equal to or higher than 1.5 standard deviations on neuropsychological tests. CN subjects must not have met criteria for MCI or dementia.

MNI

CN subjects were selected on basis of their neurological and clinical status. Initial identification of patients was based on memory complaints substantiated by an informant. A subsequent interview was conducted with a full neurological examination including the standard Mini Mental State Examination (MMSE). Routine blood screening was done to rule out underlying metabolic disorder. aMCI diagnosis was according to the Petersen criteria which included: (i) memory complaint usually corroborated by an informant; (ii) objective memory impairment for age; (iii) essentially preserved general cognitive function; (iv) largely intact functional activities; (v) not demented. Exclusion criteria included co-morbidity with other neurological disease such as stroke, Parkinson’s disease, other neurodegenerative diseases, etc; the presence of any major structural abnormalities or signs of major vascular pathology on the MRI evaluation; axis I psychiatric disorder or intellectual disability; use of psychoactive substance; previous or present use of cholinesterase inhibitor.
